# Replication efficiency of oncolytic vaccinia virus in cell cultures prognosticates the virulence and antitumor efficacy in mice

**DOI:** 10.1186/1479-5876-9-164

**Published:** 2011-09-27

**Authors:** Nanhai G Chen, Yong A Yu, Qian Zhang, Aladar A Szalay

**Affiliations:** 1 Genelux Corporation, San Diego Science Center, San Diego, CA 92109, USA; 2 Department of Radiation Oncology, Rebecca and John Moores Comprehensive Cancer Center, University of California, San Diego, CA 92093, USA; 3 Rudolph Virchow Center for Experimental Biomedicine, Department of Biochemistry and Institute for Molecular Infection Biology, University of Würzburg, D-97074 Würzburg, Germany

**Keywords:** GLV-1h68, modulation of virus replication, GI-101A tumor xenografts, oncolytic virotherapy

## Abstract

**Background:**

We have shown that insertion of the three vaccinia virus (VACV) promoter-driven foreign gene expression cassettes encoding *Renilla *luciferase-*Aequorea *GFP fusion protein, β-galactosidase, and β-glucuronidase into the *F14.5L*, *J2R*, and *A56R *loci of the VACV LIVP genome, respectively, results in a highly attenuated mutant strain GLV-1h68. This strain shows tumor-specific replication and is capable of eradicating tumors with little or no virulence in mice. This study aimed to distinguish the contribution of added VACV promoter-driven transcriptional units as inserts from the effects of insertional inactivation of three viral genes, and to determine the correlation between replication efficiency of oncolytic vaccinia virus in cell cultures and the virulence and antitumor efficacy in mice

**Methods:**

A series of recombinant VACV strains was generated by replacing one, two, or all three of the expression cassettes in GLV-1h68 with short non-coding DNA sequences. The replication efficiency and tumor cell killing capacity of these newly generated VACV strains were compared with those of the parent virus GLV-1h68 in cell cultures. The virus replication efficiency in tumors and antitumor efficacy as well as the virulence were evaluated in nu/nu (nude) mice bearing human breast tumor xenografts.

**Results:**

we found that virus replication efficiency increased with removal of each of the expression cassettes. The increase in virus replication efficiency was proportionate to the strength of removed VACV promoters linked to foreign genes. The replication efficiency of the new VACV strains paralleled their cytotoxicity in cell cultures. The increased replication efficiency in tumor xenografts resulted in enhanced antitumor efficacy in nude mice. Similarly, the enhanced virus replication efficiency was indicative of increased virulence in nude mice.

**Conclusions:**

These data demonstrated that insertion of VACV promoter-driven transcriptional units into the viral genome for the purpose of insertional mutagenesis did modulate the efficiency of virus replication together with antitumor efficacy as well as virulence. Replication efficiency of oncolytic VACV in cell cultures can predict the virulence and therapeutic efficacy in nude mice. These findings may be essential for rational design of safe and potent VACV strains for vaccination and virotherapy of cancer in humans and animals.

## Background

At least 7 million people die from cancer worldwide every year, with an estimated 12 million deaths in 2030 [1]. In the Unites States, one in two men and one in three women will be diagnosed with cancer, and one in four Americans will die from this disease [2]. Currently, the principal cancer treatment methods are surgery, radiotherapy, and chemotherapy. Although a great deal of efforts have been made to improve these conventional therapies, they are generally ineffective in treating patients with advance cancer. Thus, new treatments are urgently needed.

In the last two decades, considerable progress has been achieved in the field of oncolytic virotherapy. Oncolytic viruses have emerged as promising cancer therapeutic agents. Since the first genetically engineered oncolytic virus (oncolytic adenovirus ONYX-015) entered clinical trials in 1996, many oncolytic virus constructs from at least seven different species have been tested in a variety of clinical trials. Clinical data indicates that oncolytic viruses are generally safe in cancer patients. Furthermore, virus replication within tumors and a certain degree of therapeutic efficacy have also been reported in clinical trials [3]. Oncolytic viruses destroy tumors by several mechanisms [3], including intrinsic antitumor activity, induction of host antitumor immune responses, destruction of the tumor vasculature [4], and expression of therapeutic genes. The importance of each mechanism has yet to be determined. Oncolytic virotherapy can also effectively complement conventional cancer therapies, such as chemo- and radiotherapy.

Vaccinia virus (VACV) was used as a vaccine to eradicate smallpox that is estimated to have killed 500 million people in the 19^th ^and 20^th ^Centuries [5]. Thus, it is arguably the most successful live biotherapeutic agent. VACV is also the first oncolytic virus demonstrating viral oncolysis in the laboratory [6,7]. Many oncolytic VACVs have been tested in preclinical and clinical studies [8]. Owing to safety concerns, most of the oncolytic VACVs developed in recent years have been attenuated, mainly through viral gene inactivation. The following genes have been inactivated, either singly or in combination [8]: *F14.5L*, hemagglutinin (HA), serine protease inhibitor-1 (SPI-1), soluble type I interferon (IFN) receptor, SPI-2, thymidine kinase (TK), and vaccinia growth factor (VGF). It is noted that over-attenuation may affect antitumor efficacy. For example, a VACV triple mutant lacking SPI-1, SPI-2, and TK was reported to be greatly attenuated in mice. However, it also replicated in general less efficiently in tumor cells in culture, and its overall antitumor efficacy was lower than the parental viruses [9]. So far, the correlation between viral replication efficiency in tumor cells in culture and antitumor efficacy has not been established.

We have previously reported a light-emitting oncolytic VACV, GLV-1h68, which was constructed by inserting three foreign gene expression cassettes encoding *Renilla *luciferase-*Aequorea *green fluorescent protein fusion protein (RUC-GFP), β-galactosidase, and β-glucuronidase into the *F14.5L*, *J2R*, and *A56R *loci within the genome of VACV LIVP, respectively [10]. GLV-1h68 was greatly attenuated compared with its parent viruses [10,11]. Here, we report on the construction of a series of recombinant VACVs with fine-tuned replication efficiency by replacing one, two, or all three of these expression cassettes with short non-coding DNA sequences. We found that virus replication efficiency increased with removal of each of the expression cassettes. The increase in virus replication efficiency was proportionate to the strength of removed VACV promoters linked to foreign genes. Using this series of recombinant VACVs, we demonstrated that increased viral replication efficiency in cell cultures correlated with enhanced therapeutic efficacy, but also with increased virulence in nude mice with solid human tumor xenografts.

## Materials and methods

### Virus and cell culture

The human breast ductal adenocarcinoma cell line GI-101A [12] was kindly provided by Dr. A. Aller (Rumbaugh-Goodwin Institute for Cancer Research, Inc.) and was cultured in RPMI 1640 supplemented with 5 ng/mL of β-estradiol and progesterone (Sigma, St. Louis, CA), 10 mmol/L HEPES, 1 mmol/L sodium pyruvate, 20% fetal bovine serum (FBS; Mediatech, Inc., Manassas, VA), and 1% antibiotic-antimycotic solution (Mediatech, Inc., Manassas, VA). African green monkey kidney fibroblast CV-1 cells were purchased from American Type Culture Collection (Manassas, VA, USA) and were grown in Dulbecco's modified Eagle's medium supplemented with 10% FBS at 37°C under 5% CO_2_. GLV-1h68 was derived from VACV LIVP (Lister strain from the Institute of Viral Preparations, Moscow, Russia), as described previously [10].

### Construction of VACV shuttle vectors and generation of recombinant VACVs

The VACV *J2R *(TK) shuttle vector pCR-TKLR-gpt2 contains the left and right flanking sequences of VACV *J2R *separated by *Kpn *I, *Sac *I, and *BamH *I, and *Escherichia coli *guanine phosphoribosyltransferase (*gpt*) gene driven by the VACV early promoter p7.5 as a transient dominant selectable marker. The left flank of the TK locus in the LIVP genome was PCR amplified with the primers TKL-5 (5'-ATAAGCTTTGTTACAGATGGAAGGGTCAAA-3') and TKL-3 (5'-AGGTACCGTTTGCCATACGCTCACAGA-3') using Invitrogen High Fidelity PCR mix (Invitrogen, Carlsbad, CA). The right flanking region of the TK locus in the LIVP was PCR amplified with the primers TKR-5 (5'-TGAGCTCGGATCCTTCTGTGAGCGTATGGCAAA-3') and TKR-3 (5'-TTACTAGTACACTACGGTGGCACCATCT-3'). To construct VACV *A56R *(HA) shuttle vector, the left and right flanking sequences of VACV *A56R *were PCR-amplified from VACV LIVP using Platinum PCR SuperMix High Fidelity (Invitrogen, Carlsbad, CA) and the primers: 5'-GCGCATATGACACGATTACCAATACTTTTG-3' and 5'-GTCGGGATCCTGCGAAGCTTAGATTTCGAATACCGACGAGC-3' (left flank), 5'-GAAATCTAAGCTTCGCAGGATCCCGACTCCGGAACCAATTACTG-3' and 5'-GCGGAATTCTGATAGATTTTACTATCCCAG-3' (right flank). The two fragments were joined together using the method of gene-splicing by overlapping extension [13]. The resulting fragment was digested with *Nde *I and *EcoR *I and cloned into the same-cut pUCP7.5-gpt-1 to yield pNCVVhaT. The flanking sequences of *A56R *in the target vector were confirmed by sequencing. The VACV *F14.5L *shuttle vector pNCVVf14.5lT was constructed as described previously [11].

The recombinant viruses GLV-1h70, GLV-1h71, and GLV-1h72 were generated from the parental virus GLV-1h68 using pNCVVhaT, pNCVVf14.5lT, and pCR-TKLR-gpt2, respectively. GLV-1h73 was generated from GLV-1h70 using pNCVVf14.5lT, and GLV-1h74 from GLV-1h73 using pCR-TKLR-gpt2. All recombinant viruses were constructed using the method described previously [14].

### Viral growth curves

GI-101A cells grown in 6-well plates were infected with individual virus strains at a multiplicity of infection (MOI) of 0.01 or 10. Three wells of GI-101A cells infected individually with each virus strain were harvested at 24, 48, and 72 h post-infection (hpi). Viral particles from the infected cells were released by three cycles of freeze-thaw, followed by sonicating three times for 1 min at full power using the Branson sonifier 450 before titration. Virus strains were titrated in CV-1 cells in duplicates.

### Cytotoxicity assays in cell culture

Cytotoxcity was performed as described previously [14]. Briefly, GI-101A cells were plated at 2 × 10^4 ^cells per well in 96-well plates and incubated at 37°C in a CO_2 _incubator overnight. Cells were either mock-infected or infected with each virus strain at an MOI of 0.01. Viral cytotoxity was assayed daily for 5 d. Lactate dehydrogenase (LDH) activity was quantified using a CytoTox 96 Non-Radioactive Cytotoxicity Assay kit (Promega, Madison, WI). Results are expressed as the percentage of surviving cells. This percentage was determined using the formula: (cell lysate-supernatant)_tx_/(cell lysate-supernatant)_t0 _× 100. In the formula, (cell lysate-supernatant)_tx _and (cell lysate-supernatant)_t0 _represent LDH activity in the cells after and before infection, respectively.

### Virus virulence and tumor therapy in mice

All mice were cared for and maintained in accordance with animal welfare regulations under an approved protocol by the Institutional Animal Care and Use Committee of Explora Biolabs (San Diego Science Center, San Diego, CA). GI-101A xenograft tumors were developed in 6- to 8-week-old male nude mice (NCI:Hsd:Athymic Nude-*Foxn1*^nu^, Harlan) by implanting 5 × 10^6 ^GI-101A cells subcutaneously on the right hind leg. Tumor growth was recorded once a week in three dimensions using a digital caliper. Tumor volume was calculated as ([length × width × height]/2) and reported in mm^3^. Thirty-three days after tumor cell implantation, mice were injected with a single intravenous dose of 5 × 10^6 ^plaque forming units (pfu) of individual virus strains in 100 μL of PBS. Animals were observed daily for any sign of virulence. Fourteen days post virus injection, four animals from each group were sacrificed for analysis of virus titers in tumors as described previously [10].

### Statistical analysis

Statistical analyses were performed with GraphPad Prism, version 5.03 (GraphPad Software Inc., San Diego, CA). Comparisons of treatment groups were made by either unpaired t test or two-way analysis of variance (ANOVA) with Bonferroni comparison post-tests as indicated in the figure legends. The post-test was only performed when ANOVA revealed significance. Statistical analysis of survival was assessed using the log-rank test. Values of *P *less than 0.05 were considered significant.

## Results

### Generation of the derivatives of GLV-1h68 by replacing the foreign gene expression cassettes with short non-coding DNA sequences

**Figure 1:**
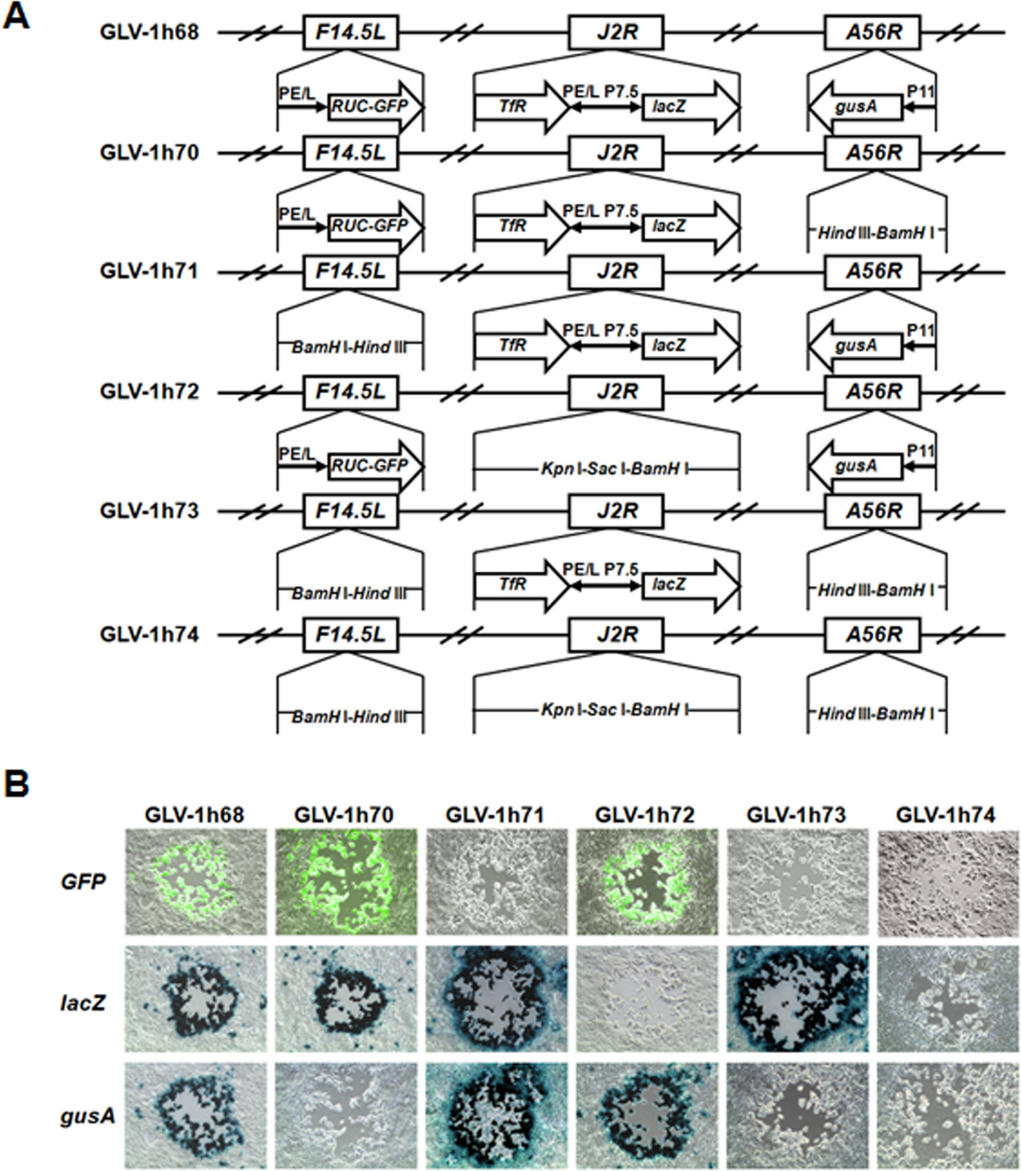
**GLV-1h68 and its derivatives**. (A) Schematic representation of the genomic structures of the recombinant vaccinia virus GLV-1h68 and its marker gene expression cassette removal derivatives. PE/L, P11, and P7.5 are VACV synthetic early/late, 11K, and 7.5K promoters, respectively. *TfR *is human transferin receptor inserted in the reverse orientation with respect to the promoter PE/L. (B) Marker gene expression and genotype verification. CV-1 cells were infected with each individual virus strain. Two days post-infection, the GFP expression was visualized by fluorescence microscopy and expression of β-galactosidase and β-glucuronidase was detected by X-gal and X-GLcA staining, respectively.

Previously, we have shown that insertion of the foreign marker gene expression cassettes into the *F14.5L*, *J2R *(encoding thymidine kinase, TK), and *A56R *(encoding hemagglutinin, HA) loci of the VACV LIVP genome disrupted the open-reading frames (ORFs) *F14.5L*, *J2R*, and *A56R*, resulting in the recombinant VACV GLV-1h68 with enhanced tumor colonization specificity as well as reduced virulence in mice [10,11]. To determine the contributions of individual foreign gene expression to virus attenuation, in addition to viral gene inactivation, we constructed a series of the derivatives of GLV-1h68 by replacing one, two, or all three of these expression cassettes in the genome of GLV-1h68 with short non-coding DNA sequences while the original ORFs *F145L*, *J2R*, and *A56R *remain disrupted in all the derivatives. The RUC-GFP expression cassette at the *F14.5L *locus was replaced with the short DNA sequence "GGATCCTGCGAAGCTT" comprising *BamH *I and *Hind *III sites (underlined). The foreign inserts (human transferin receptor and *lac*Z) at the *J2R *locus were replaced with the non-coding DNA sequence "CTGTGAGCGTATGGCAAACGGTACCGAGCTCGGATCC" consisting of *Kpn *I, *Sac *I, and *BamH *I sites. The β-glucuronidase expression cassette at the *A56R *locus was replaced with the short DNA sequence "TAAGCTTCGCAGGATCCC" comprising *Hind *III and *BamH *I sites. The genotype of each derivative shown in Figure 1A was verified by PCR, followed by DNA sequencing. The status of expression of GFP, β-galactosidase, and β-glucuronidase by each derivative was confirmed by fluorescence microscopy, 5-bromo-4-chloro-3-indolyl-β-D-galactopyranoside (X-gal), or 5-bromo-4-chloro-3-indolyl-β-D-glucuronic acid (X-GlcA) staining, respectively (Figure 1B).

### Excision of the foreign expression cassettes from GLV-1h68 enhances virus replication efficiency in cell cultures

**Figure 2:**
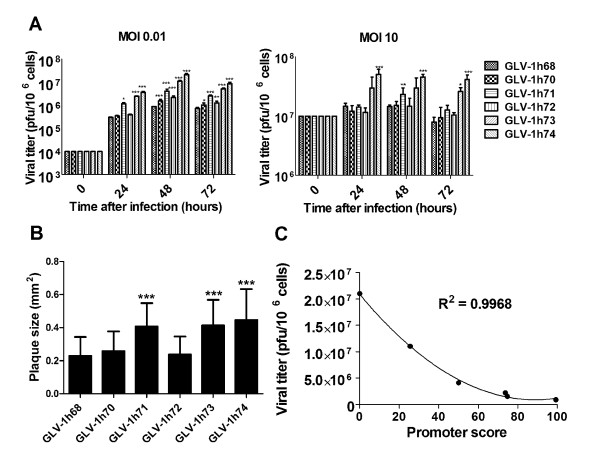
**Excision of the foreign expression cassettes from GLV-1h68 enhances virus replication efficiency**. (A) Viral growth curves. GI-101A cells were infected with GLV-1h68 or its derivatives at an MOI of 0.01 or 10, and harvested at 24, 48, and 72 hpi. Viral titers were determined in CV-1 cells. The values are the mean of triplicate samples, and the bars indicate SD. The data represents two independent experiments. Statistical analysis was performed using two-way ANOVA. *, **, and *** indicate *P *< 0.05, 0.01, and 0.001, respectively, when compared with the GLV-1h68 group. (B) Plaque size comparison. Human breast tumor GI-101A cells were infected with 200 pfu of each virus strain for three days, and stained with crystal violet. The values are the mean of more than 58 plaques, and the bars indicate SD. Representative of two independent experiments. Statistical analysis was performed using unpaired t-test. GLV-1h71, GLV-1h73, and GLV-1h74 formed significantly larger plaques then did GLV-1h68. *** indicates *P *< 0.001 when compared to GLV-1h68. (C) Correlation between the promoter scores of GLV-1h68 and its derivative with their virus yields in the GI-101A cell culture (48 hpi, MOI 0.01).

To examine whether removal of any of the originally introduced foreign gene expression cassettes from GLV-1h68 would affect virus replication, human breast tumor GI-101A cells in culture were infected with the parental virus GLV-1h68 and its derivatives at an MOI of 0.01 or 10. The infected cells were harvested at 24, 48, and 72 hpi and the viral titers at each time point determined in CV-1 cells using standard plaque assays. At the MOI of 0.01, GLV-1h71 (RUC-GFP^-^), GLV-1h73 (RUC-GFP^-^*/gus*A^-^), and GLV-1h74 (RUC-GFP^-^/*lac*Z^-^/*gus*A^-^) showed significantly enhanced virus replication at all time points examined, whereas GLV-1h70 (*gus*A^-^) and GLV-1h72 (*lac*Z^-^) demonstrated significantly better virus replication at 48 and 72 hpi, but not at 24 hpi, compared with the parent virus GLV-1h68 (Figure 2A). At the MOI of 10, GLV-1h71 (RUC-GFP^-^), GLV-1h73 (RUC-GFP^-^*/gus*A^-^), and GLV-1h74 (RUC-GFP^-^/*lac*Z^-^/*gus*A^-^) showed significantly higher replication efficiency at 48h, 72h, and all time points, respectively, than GLV-1h68 (Figure 2A). The difference in virus replication among all the viruses was more prominent at the low MOI (0.01) than that at the high MOI (10). Furthermore, GLV-1h71 (RUC-GFP^-^), GLV-1h73 (RUC-GFP^-^*/gus*A^-^), and GLV-1h74 (RUC-GFP^-^/*lac*Z^-^/*gus*A^-^) formed significantly larger plaques than GLV-1h68 in GI-101A cells (Figure 2B). The plaques formed by GLV-1h70 (*gus*A^-^) and GLV-1h72 (*lac*Z^-^) were consistently slightly larger than that formed by GLV-1h68. However, the differences in plaque size among GLV-1h68, GLV-1h70 (*gus*A^-^), and GLV-1h72 (*lac*Z^-^) were not statistically significant. Taken together, removal of any of the foreign expression cassettes or in combination, enhanced virus replication in each cell lines, irrespective of which foreign gene was removed. Among three foreign expression cassettes within the genome of GLV-1h68, removal of the RUC-GFP expression cassette had the greatest enhanced effect on virus replication. GLV-1h73 (RUC-GFP^-^*/gus*A^-^) with two of the foreign gene expression cassettes removed, replicated more rapidly than any of the single foreign gene expression cassette removal viruses, whereas GLV-1h74 (RUC-GFP^-^/*lac*Z^-^/*gus*A^-^) in which all three foreign expression cassettes were replaced, demonstrated the highest replication efficiency among all the virus strains tested. Thus, a series of recombinant VACV strains with fine-tuned replication efficiency were generated by replacing one, two, or three expression cassettes in the genome of GLV-1h68 with short non-coding DNA sequences.

### Replication efficiency of GLV-1h68 and its derivatives in cell cultures inversely correlates with added strength of the inserted promoters remaining in each viral genome

As demonstrated above, replication efficiency of GLV-1h68 and its derivatives in cell cultures was inversely proportionate with the number of foreign gene expression cassettes present in the viral genome. Among the single foreign gene expression cassette removal derivatives, GLV-1h71 (RUC-GFP^-^) showed the highest replication efficiency. It is interesting to note that the RUC-GFP expression cassette that was replaced in GLV-1h71 contains a synthetic early/late promoter (PE/L), the strongest promoter among the promoters introduced into the genome of GLV-1h68 [15]. It is likely that the extra transcriptional and translational burden imposed by over-expression of the foreign gene expression cassettes might slow down virus replication. Since an extra transcriptional and translational burden is directly related to the strength of a promoter, we sought to analyze the relationship between the replication efficiency of the GLV-1h68 derivatives and the strength of the inserted promoters remaining in each viral genome. Firstly, we assigned an index to each promoter according to their strength as reported before [15]. The index of the VACV P7.5 K promoter (P7.5) was set to 1, and the indices of the VACV P11K promoter (P11) and PE/L were 24.5 and 49.2, respectively, since P11 and PE/L were reported to be 24.5 and 49.2 times stronger than P7.5, respectively [15]. The index of PE/L at the *J2R *locus was half of the index of the PE/L at the *F14.5L *locus (24.6) since the gene encoding human transferring receptor was inserted in the reverse orientation with respect to the promoter. Secondly, the promoter scores for GLV-1h68 and its derivatives were calculated by adding together the index of each inserted promoter remaining in each virus strain. The resulting promoter scores for GLV-1h68, GLV-1h70 (*gus*A^-^), GLV-1h71 (RUC-GFP^-^), GLV-1h72 (*lac*Z^-^), GLV-1h73 (RUC-GFP^-^*/gus*A^-^), and GLV-1h74 (RUC-GFP^-^/*lac*Z^-^/*gus*A^-^) were 99.3, 74.8, 50.1, 73.7, 25.6, and 0, respectively. Regression analysis indicated that there was an inverse correlation between the promoter scores and the virus replication efficiency in the GI-101A cell culture with a value of R^2 ^being 0.9968 (Figure 2C). Thus, replication efficiency of GLV-1h68 and its derivatives in the cell culture inversely correlated with added strength of the inserted promoters remaining in each viral genome.

### Foreign gene expression cassette removal derivatives kill tumor cells in culture more efficiently than their parental virus GLV-1h68

**Figure 3:**
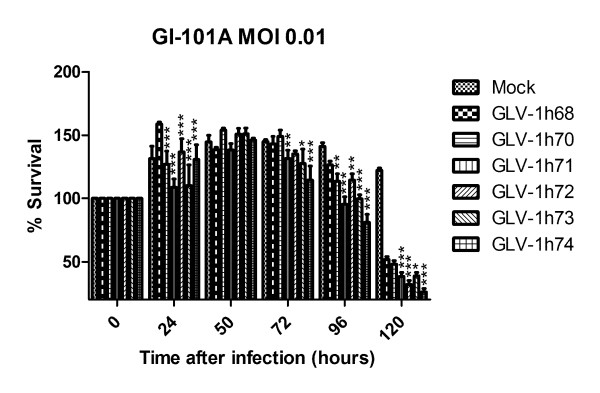
**Foreign gene expression cassette removal derivatives kill tumor cells in culture more efficiently than their parental virus GLV-1h68**. GI-101A cells were infected with each virus strain at an MOI of 0.01. Cell viability was determined using CytoTox 96 Non-Radioactive Cytotoxicity Assay kit (Promega, Madison, WI, USA). Cell survival before infection was set at 100%. The values are the mean of quadruplicate samples, and the bars indicate SD. The data represents two independent experiments. Statistical analysis was performed using two-way ANOVA. *, **, and *** indicate *P *< 0.05, 0.01, and 0.001, respectively, when compared with the GLV-1h68 group.

After demonstrating that vaccinia virus replication efficiency in cell cultures was inversely proportionate to the number of foreign gene expression cassettes present within the viral genome and inversely correlated with the strength of promoters inserted into each viral genome, we compared the tumor cell-killing potential of each virus strain. Human breast tumor GI-101A cells were infected with each virus strain at an MOI of 0.01, and cell viability was measured daily for 5 d after infection. In general, the number of viable cells continued to increase until 2d after infection owing to the fact that only a small portion of cells was initially infected at the low MOI. Compared with mock infection, GLV-1h68 infection resulted in a significant decrease in the number of viable cells at 96 and 120 hpi (*P *< 0.01, 0.001, respectively). All foreign gene expression cassette removal derivatives showed significantly more efficient tumor cell killing than their parent virus GLV-1h68 at certain time points after infection (Figure 3). There was a general trend that virus strains with higher replication efficiency also killed cancer cells more efficiently. Thus, virus replication efficiency was in accord with its tumor cell-killing capacity.

### Replication efficiency of GLV-1h68 and its derivatives in tumor xenografts positively correlates with their replication efficiency in cell cultures

**Figure 4:**
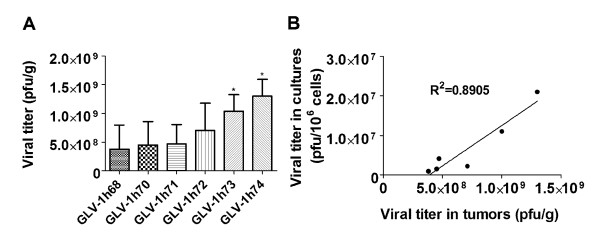
**Virus growth in GI-101A tumor xenografts positively correlates with *v*irus yields in cell cultures**. (A) Viral titers in the GI-101A tumors. Nude mice bearing the GI-101A tumors were injected i.v. with each virus strain at 5 × 10^6 ^pfu/mouse. Tumors were harvested at 14 days post virus administration. The values are the mean of quadruplicate samples, and the bars indicate SD. Statistical analysis was performed using unpaired t test with Welch's correction. * indicates *P *< 0.05, when compared with the GLV-1h68 group. (B) Correlation between virus yields in the GI-101A cell culture (48 hpi, MOI 0.01) and virus growth in the GI-101A tumors.

To compare replication efficiency of GLV-1h68 and each of its derivatives in tumor xenografts, nude mice bearing the GI-101A tumors were injected intravenously (i.v.) with each virus strain at 5 × 10^6 ^pfu per mouse. Tumors were harvested at 14 days post virus administration. Viral titers in tumors were determined in CV-1 cells using standard plaque assays. As shown in Figure 4A, GLV-1h73 (RUC-GFP^-^*/gus*A^-^) and GLV-1h74 (RUC-GFP^-^/*lac*Z^-^/*gus*A^-^) with two and three of the foreign expression cassettes removed, respectively, gave significantly higher virus yields than their parental virus GLV-1h68. Although there was no statistically significant difference in virus yield between GLV-1h68 and single cassette-replaced virus strains, these virus strains consistently gave higher virus yields than GLV-1h68 in the GI-101A and other different tumor models tested (Figure 4A). Regression analysis indicated that there was a linear correlation between virus yields in the GI-101A tumors and that in the GI-101A cell culture (Figure 4B).

### Enhanced virus replication efficiency predicts enhanced antitumor efficacy

**Figure 5:**
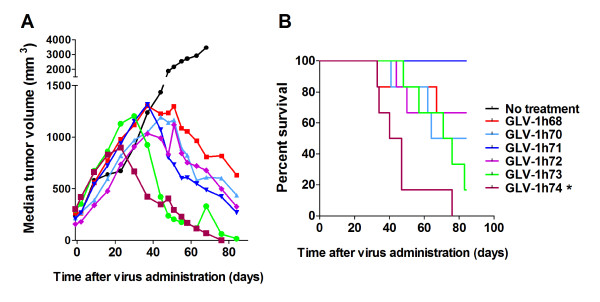
**Virus replication efficiency in cell cultures predicts antitumor efficacy and virulence in nude mice with tumor xenografts**. Tumor-bearing mice (n = 6) were injected i.v. with each virus strain at a single dose of 5 × 10^6 ^pfu/mouse at 34 days after tumor cell implantation. Tumor volume (A) and mouse survival (B) were monitored for 84 days after virus administration. Statistical analysis of the survival data was performed using the log-rank test. * indicates *P *< 0.05, when compared with the GLV-1h68 group. All untreated mice were sacrificed 102 days after tumor cell implantation (equivalent to 68 days post treatment for the treated groups).

After demonstrating that removal of the marker gene expression cassettes enhanced virus replication and tumor cell killing in cell cultures as well as virus replication in tumors, we sought to determine if enhanced virus replication could be translated into improved therapeutic efficacy. Nude mice bearing the GI-101A tumors of 200-300 mm^3 ^were injected i.v. with each virus strain at a single dose of 5 × 10^6 ^pfu/mouse. Tumor volume was monitored weekly for 84 days. As depicted in Figure 5A, not surprisingly, GLV-1h68 efficiently shrank the GI-101A tumors as demonstrated before [10]. All the derivatives showed enhanced tumor-shrinking capability compared with the parental virus GLV-1h68. Strikingly, tumors treated with GLV-1h74 (RUC-GFP^-^/*lac*Z^-^/*gus*A^-^) with all of the foreign expression cassettes excised, stopped growing three weeks after virus administration, which was two weeks earlier than the date when GLV-1h68-treated tumors ceased to grow. Overall, virus strains that replicated more efficiently in the GI-101A cell culture, also shank the GI-101A tumors faster.

### Enhanced virus replication efficiency predicts increased virulence in nude mice

To evaluate if removal of the foreign gene expression cassettes from GLV-1h68 would affect virus virulence in mice, groups of 6 nude mice bearing the GI-101A tumors were treated with each virus strain individually as described above for the efficacy study (untreated group had 4 mice). Mouse survival was monitored daily. Due to excessive tumor burden, all untreated mice were sacrificed 102 days after tumor cell implantation (equivalent to 68 days post treatment for the treated groups). All treated groups were observed for 84 days after virus treatment. During this period, all mice treated with GLV-1h71 (RUC-GFP^-^) survived and looked healthy. Two mice each treated with GLV-1h68 or GLV-1h72 (*lac*Z^-^) died. Three, 5, and all 6 mice treated with GLV-1h70 (*gus*A^-^), GLV-1h73 (RUC-GFP^-^*/gus*A^-^), and GLV-1h74 (RUC-GFP^-^/*lac*Z^-^/*gus*A^-^) died, respectively. Statistically, there was a significant difference in survival between the GLV-1h68 treated group and the GLV-1h74-treated group, but no significant difference between GLV-1h68 treated group and any other virus-treated groups (Figure 5B). Thus, excision of one of the foreign expression cassettes from GLV-1h68 seemed not to significantly affect virus virulence, whereas removal of all of three foreign expression cassettes significantly enhanced virus virulence. Although there was no significant difference in survival between GLV-1h68 treated group and GLV-1h73 (RUC-GFP^-^*/gus*A^-^) treated group, we consistently observed higher virulence associated with GLV-1h73 (RUC-GFP^-^*/gus*A^-^) than GLV-1h68 in additional tumor models. Overall, virus virulence in tumorous mice directly reflected the replication efficiency of these virus strains in cell cultures.

## Discussion

GLV-1h68, one of the most widely studied oncolytic VACVs, has been shown to be effective in treating large numbers of human and canine cancers in preclinical studies [10,16-24]. It is currently being evaluated in phase I/II clinical trials http://www.clinicaltrials.gov. GLV-1h68 was derived from the wild-type VACV LIVP by insertional inactivation of the ORFs *F14.5L*, *J2R*, and *A56R *[10]. Our previous studies demonstrated that GLV-1h68 was much attenuated in nude mice compared with its parental virus strains [10,11]. Inactivation of *F14.5L*, *J2R*, and *A56R *was previously shown to reduce VACV virulence in mice [11,25-27]. In this study, we decided to determine how much the expression of foreign marker genes, in addition to viral gene interruption, contributed to the level of attenuation of GLV-1h68. When an individual foreign gene expression cassette was replaced with a short non-coding DNA sequence, replacement of any single expression cassettes resulted in elevated replication in cell cultures compared to their parent virus GLV-1h68. Replacement of the RUC-GFP expression cassette resulted in the most enhanced virus replication, probably owing to the fact that the synthetic early/late promoter that drives RUC-GFP expression is the strongest in comparison with the other promoters [15]. GLV-1h73 (RUC-GFP^-^*/gus*A^-^) with two of the foreign gene expression cassettes removed replicated more efficiently than any of the virus strains with single foreign gene expression cassette removed. GLV-1h74 (RUC-GFP^-^/*lac*Z^-^/*gus*A^-^) in which all three foreign expression cassettes were replaced, demonstrated the highest replication efficiency among all the virus strains tested. Interestingly, we have noticed that replication efficiency of GLV-1h68 and its foreign gene removal derivatives was inversely proportionate to the added strength of the promoters present at the loci of *F14.5L*, *J2R*, and *A56R *in each virus strain. Thus, the expression of the foreign genes driven by the VACV promoters that adds an extra transcriptional and translational burden onto the virus and infected cells contributed to the attenuation of GLV-1h68 replication. Since the degree of attenuation of VACV strains is proportionate to the strength of promoters that drive foreign gene expression, therefore, it may be possible to fine tune VACV replication efficiency by choosing VACV promoters of appropriate strength to facilitate foreign gene expression in VACV as was demonstrated for the first time in this study.

The mechanisms by which oncolytic viruses destroy tumors are currently under intense investigation. It is believed that an unarmed oncolytic virus destroys tumors through one, or most likely, a combination of the following mechanisms: 1) by direct viral oncolysis of tumor cells; 2) by destruction of the tumor vasculature [4,28]; and 3) by induction of host antitumoral immune responses [29,30]. However, importance of each mechanism is still a matter of controversy, and might be dependent on types of tumors, viruses, and hosts studied. Kirn *et al. *reported that a VACV WR-derived oncolytic virus infected tumor-associated vascular endothelial cells, resulting in vascular collapse in infected tumors [31]. In contrast, we recently showed that the oncolytic VACV GLV-1h68 did not destroy endothelial cells in tumors. The tumor vasculature in infected tumors was still functional [32]. These differences in the results might reflect differences in the cell tropisms of VACV WR and LIVP derived virus strains, and/or might be tumor type dependent. In a study investigating the role of antitumor immunity in comparison to the role of direct oncolysis in reovirus-mediated virotherapy, Prestwich *et al. *found that the immune response, but not direct viral oncolysis or replication, was a critical factor for reovirus-mediated tumor rejection or therapy [33]. Previously, we reported massive VACV-mediated intratumoral inflammations in GLV-1h68-infected tumors [10]. However, following studies indicated that VACV-mediated immune responses were not essential for tumor elimination [32]. Instead, viral-mediated direct tumor cell killing seems to be a key factor for GLV-1h68-mediated oncolytic virotherapy, suggesting that enhanced virus replication efficiency and viral spreading within the tumor tissue may drastically improve therapeutical outcome. In this study, we generated a series of recombinant VACVs with different replication efficiency, which allowed us to evaluate the correlation between virus replication efficiency and therapeutical efficacy. The results described here indicated that enhanced virus replication efficiency resulted in enhanced tumor cell killing in cell cultures and similarly enhanced virus replication in tumor xenografts, yielding enhanced therapeutical efficacy. These data further strengthen previous reports that viral oncolysis is the most critical in tumor rejection and elimination [32].

Safety is a major concern for any cancer therapeutics. During the past years, strategies have been developed to enhance tumor selectivity and clinical safety of oncolytic viruses [3]. These strategies include: 1) inactivation of viral genes; 2) transcriptional targeting; 3) regulation of mRNA stability; 4) mRNA translational control; and 5) transductional targeting. Inactivation of viral genes is the main strategy currently used to enhance tumor specific replication of VACV strains [8]. However, it was reported that an increase in attenuation resulted in a decrease in therapeutic efficacy [9]. Therefore, it is important to fine balance virulence and efficacy. GLV-1h68 was shown to be safe in numerous tumor xenograft models. Here, we showed that removal of the foreign expression cassettes from GLV-1h68 resulted in increased virulence in mice. Although removal of one of the three inserted foreign gene expression cassettes seemed not to significantly enhance virus virulence, removal of two of the three foreign gene expression cassettes simultaneously resulted in consistently increased virus virulence. Removal of all three foreign gene expression cassettes at the same time resulted in a virus strain with a significant increase in virus virulence. The foreign gene expression cassettes inserted into GLV-1h68 seems to help limit virus virulence in mice.

## Conclusions

Taken together, we have shown that virus promoter driven expression of foreign genes did in fact attenuate VACV replication. We found that virus replication efficiency was inversely proportionate to the added strength of promoters linked to foreign genes inserted into the viral genome. Thus, we propose that VACV replication can be fine tuned through carefully choosing the strength of promoters added to the VACV genome for the expression of foreign genes. Furthermore, VACV replication efficiency in cell cultures paralleled cytotoxicity in cell cultures, and importantly, replication efficiency in tumors as well as therapeutic efficacy in nude mice. However, enhanced virus replication efficiency in cell cultures resulted in increased virulence in mice.

In conclusion, GLV-1h68 seems to have well balanced replication efficiency and minimum virulence in mice to achieve highly efficacious tumor rejection in nude mice with human xenografts. Virus strain attenuation by insertion of additional viral promoters of different strength into the VACV genome may result in rationally designed safe attenuated therapeutic strains, presumably due to competition for VACV RNA polymerase and cellular translation machinery required to facilitate transcription and translation of viral genes.

## Competing interests

Nanhai G. Chen, Yong A. Yu, Qian Zhang, and Aladar A. Szalay are affiliated with Genelux Corporation.

## Authors' contributions

NGC participated in the design of study, carried out the experiments including construction of the *F14.5L*, *A56R *shuttle vectors, generation of the recombinant vaccinia viruses, virus replication assays, cytotoxicity assays, and virus titrations, performed the statistical analysis and interpretation of data, and drafted the manuscript. YAY was involved in the animal studies. QZ constructed the *J2R *shuttle vector. AAS conceived of the study, participated in its design and coordination, and prepared the final version of manuscript. All authors read and approved the final manuscript.

## References

[B1] Cancer fact sheet http://www.who.int/mediacentre/factsheets/fs297

[B2] Cancer Facts & Figures 2010 http://www.cancer.org/acs/groups/content/@nho/documents/document/acspc-024113.pdf

[B3] Chen NG Szalay AA Minev BR Oncolytic virotherapy of cancer Cancer Managment in Man: Chemotherapy, Biological Therapy, Hyperthermia and Supporting Measures 2011 13 New York: Springer 295 316 [Nasir A, Yeatman TJ (Series Editor) *Cancer Growth and Progression*]

[B4] Kirn DH Thorne SH Targeted and armed oncolytic poxviruses: a novel multi-mechanistic therapeutic class for cancer Nat Rev Cancer 2009 9 64 71 10.1038/nrc2545 19104515

[B5] Fenner F Henderson DA Arita I Jezek Z Ladnyi ID Smallpox and its eradication 1988 Geneva: World Health Organization

[B6] Levaditi C Nicolau S Sur le culture du virus vaccinal dans les neoplasmes epithelieux CR Soc Biol 1922 86 928

[B7] Southam CM Present status of oncolytic virus studies Trans N Y Acad Sci 1960 22 657 673 13833074 10.1111/j.2164-0947.1960.tb00739.x

[B8] Chen NG Szalay AA Oncolytic vaccinia virus: a theranostic agent for cancer Future Virol 2010 5 763 784 10.2217/fvl.10.58

[B9] Yang S Guo ZS O'Malley ME Yin X Zeh HJ Bartlett DL A new recombinant vaccinia with targeted deletion of three viral genes: its safety and efficacy as an oncolytic virus Gene Ther 2007 14 638 647 10.1038/sj.gt.3302914 17268533

[B10] Zhang Q Yu YA Wang E Chen N Danner RL Munson PJ Marincola FM Szalay AA Eradication of solid human breast tumors in nude mice with an intravenously injected light-emitting oncolytic vaccinia virus Cancer Res 2007 67 10038 10046 10.1158/0008-5472.CAN-07-0146 17942938

[B11] Zhang Q Liang C Yu YA Chen N Dandekar T Szalay AA The highly attenuated oncolytic recombinant vaccinia virus GLV-1h68: comparative genomic features and the contribution of F14.5L inactivation Mol Genet Genomics 2009 282 417 435 10.1007/s00438-009-0475-1 19701652 PMC2746888

[B12] Rathinavelu P Malave A Raney SR Hurst J Roberson CT Rathinavelu A Expression of mdm-2 oncoprotein in the primary and metastatic sites of mammary tumor (GI-101) implanted athymic nude mice Cancer Biochem Biophys 1999 17 133 146 10738909

[B13] Horton RM Ho SN Pullen JK Hunt HD Cai Z Pease LR Gene splicing by overlap extension Methods Enzymol 1993 217 270 279 8474334 10.1016/0076-6879(93)17067-f

[B14] Chen N Zhang Q Yu YA Stritzker J Brader P Schirbel A Samnick S Serganova I Blasberg R Fong Y Szalay AA A novel recombinant vaccinia virus expressing the human norepinephrine transporter retains oncolytic potential and facilitates deep-tissue imaging Mol Med 2009 15 144 151 19287510 10.2119/molmed.2009.00014 PMC2654849

[B15] Chakrabarti S Sisler JR Moss B Compact, synthetic, vaccinia virus early/late promoter for protein expression Biotechniques 1997 23 1094 1097 9421642 10.2144/97236st07

[B16] Lin SF Yu Z Riedl C Woo Y Zhang Q Yu YA Timiryasova T Chen N Shah JP Szalay AA Treatment of anaplastic thyroid carcinoma in vitro with a mutant vaccinia virus Surgery 2007 142 976 983 discussion 976-983 10.1016/j.surg.2007.09.017 18063085

[B17] Kelly KJ Woo Y Brader P Yu Z Riedl C Lin SF Chen N Yu YA Rusch VW Szalay AA Fong Y Novel oncolytic agent GLV-1h68 is effective against malignant pleural mesothelioma Hum Gene Ther 2008 19 774 782 10.1089/hum.2008.036 18754710 PMC2940611

[B18] Lin SF Price DL Chen CH Brader P Li S Gonzalez L Zhang Q Yu YA Chen N Szalay AA Oncolytic vaccinia virotherapy of anaplastic thyroid cancer in vivo J Clin Endocrinol Metab 2008 93 4403 4407 10.1210/jc.2008-0316 18697871 PMC3728375

[B19] Gentschev I Stritzker J Hofmann E Weibel S Yu YA Chen N Zhang Q Bullerdiek J Nolte I Szalay AA Use of an oncolytic vaccinia virus for the treatment of canine breast cancer in nude mice: preclinical development of a therapeutic agent Cancer Gene Ther 2009 16 320 328 10.1038/cgt.2008.87 18949014

[B20] Yu YA Galanis C Woo Y Chen N Zhang Q Fong Y Szalay AA Regression of human pancreatic tumor xenografts in mice after a single systemic injection of recombinant vaccinia virus GLV-1h68 Mol Cancer Ther 2009 8 141 151 10.1158/1535-7163.MCT-08-0533 19139123 PMC2664310

[B21] Yu Z Li S Brader P Chen N Yu YA Zhang Q Szalay AA Fong Y Wong RJ Oncolytic vaccinia therapy of squamous cell carcinoma Mol Cancer 2009 8 45 19580655 10.1186/1476-4598-8-45 PMC2714037

[B22] Gentschev I Donat U Hofmann E Weibel S Adelfinger M Raab V Heisig M Chen N Yu YA Stritzker J Szalay AA Regression of human prostate tumors and metastases in nude mice following treatment with the recombinant oncolytic vaccinia virus GLV-1h68 J Biomed Biotechnol 2010 2010 489759 20379368 10.1155/2010/489759 PMC2850154

[B23] Gentschev I Ehrig K Donat U Hess M Rudolph S Chen N Yu YA Zhang Q Bullerdiek J Nolte I Significant Growth Inhibition of Canine Mammary Carcinoma Xenografts following Treatment with Oncolytic Vaccinia Virus GLV-1h68 J Oncol 2010 2010 736907 20631910 10.1155/2010/736907 PMC2902752

[B24] Seubert CM Stritzker J Hess M Donat U Sturm JB Chen N von Hof JM Krewer B Tietze LF Gentschev I Szalay AA Enhanced tumor therapy using vaccinia virus strain GLV-1h68 in combination with a beta-galactosidase-activatable prodrug seco-analog of duocarmycin SA Cancer Gene Ther 2011 18 42 52 10.1038/cgt.2010.49 20829890 PMC3007590

[B25] Izmailyan R Chang W Vaccinia virus WR53.5/F14.5 protein is a new component of intracellular mature virus and is important for calcium-independent cell adhesion and vaccinia virus virulence in mice J Virol 2008 82 10079 10087 10.1128/JVI.00816-08 18684811 PMC2566269

[B26] Buller RM Chakrabarti S Cooper JA Twardzik DR Moss B Deletion of the vaccinia virus growth factor gene reduces virus virulence J Virol 1988 62 866 874 3339716 10.1128/jvi.62.3.866-874.1988 PMC253644

[B27] Shida H Hinuma Y Hatanaka M Morita M Kidokoro M Suzuki K Maruyama T Takahashi-Nishimaki F Sugimoto M Kitamura R Effects and virulences of recombinant vaccinia viruses derived from attenuated strains that express the human T-cell leukemia virus type I envelope gene J Virol 1988 62 4474 4480 3184271 10.1128/jvi.62.12.4474-4480.1988 PMC253556

[B28] Breitbach CJ De Silva NS Falls TJ Aladl U Evgin L Paterson J Sun YY Roy DG Rintoul JL Daneshmand M Targeting Tumor Vasculature With an Oncolytic Virus Mol Ther 2011 886 94 10.1038/mt.2011.26 PMC3098639 21364541

[B29] Prestwich RJ Errington F Diaz RM Pandha HS Harrington KJ Melcher AA Vile RG The case of oncolytic viruses versus the immune system: waiting on the judgment of Solomon Hum Gene Ther 2009 20 1119 1132 10.1089/hum.2009.135 19630549 PMC2829276

[B30] Parato KA Lichty BD Bell JC Diplomatic immunity: turning a foe into an ally Curr Opin Mol Ther 2009 11 13 21 19169955

[B31] Kirn DH Wang Y Le Boeuf F Bell J Thorne SH Targeting of interferon-beta to produce a specific, multi-mechanistic oncolytic vaccinia virus PLoS Med 2007 4 e353 10.1371/journal.pmed.0040353 18162040 PMC2222946

[B32] Weibel S Raab V Yu YA Worschech A Wang E Marincola FM Szalay AA Viral-mediated oncolysis is the most critical factor in the late-phase of the tumor regression process upon vaccinia virus infection BMC Cancer 2011 11 68 10.1186/1471-2407-11-68 21320309 PMC3044654

[B33] Prestwich RJ Ilett EJ Errington F Diaz RM Steele LP Kottke T Thompson J Galivo F Harrington KJ Pandha HS Immune-mediated antitumor activity of reovirus is required for therapy and is independent of direct viral oncolysis and replication Clin Cancer Res 2009 15 4374 4381 10.1158/1078-0432.CCR-09-0334 19509134 PMC4821072

